# The Book World of Medicine and Science

**Published:** 1899-07-29

**Authors:** 


### The Book World of Medicine and Science.

The Anatomy of the Central Nervous System of Man, and of Vertebrates in General. By Professor Ludwig Edinger, M.D. Translated by Winfield S. Hall, Ph.D., &c., from the 5th German edition. (Philadelphia and New York: The F. A. Davis Company, 1899. Pp. 446. 258 engravings).

This magnificent work is a truly remarkable proof of the careful and laborious attention that is now paid to facts having the remotest bearings on pathological problems. It is a work worthy to rank as an anatomical complement to Sir William Gower's brilliant treatise, a book to which, in its breadth, accuracy, and lucidity, it bears much resemblance. Perhaps one of the greatest merits of Edinger's volume is that it discusses the anatomy of man's central nervous system in the light of embryology and the teachings of comparative anatomy. And further, the subject is introduced by an admirable historical *résumé* and a philosophic chapter on fundamental conceptions. Although, of course, structure—macroscopical and microscopical—is the subject of the book, function is not neglected, as it is in the works of more narrow-minded anatomists. It would be a little ungracious to pick holes in such a book as this, yet necessarily, dealing as it does with so vast a collection of facts, with the work of so many authors, there are some weak points. We miss some reference to Dr. Gaskell's well-known theories, and the later work of English neurologists generally seems perhaps unduly neglected. The name of Horsley occurs but once, and other names of note are to be sought in vain. Perhaps the weakest portion of the book is that dealing with the brain cortex; a little more physiological leaven would have been welcomed. An English reader will perh aps at first find some difficulty in following the use of the terms "dendrites," "neurexons," and some others. But the translator has been careful to set out in footnotes the exact conceptions which are connoted by these terms in this book. The translator, assisted by Messrs. Holland and Carlton, has done his work well, and employed a clear, vigorous, and lucid style. The diagrams are good. In fact the whole work is a monument to German science and American scholarship.

Claude Bernard. By Michael Foster, M.A., M.D., D.C.L., &c. (London: T. Fisher Unwin. 1899. Price 3s. 6d.)

The book before us is a further instalment of the Masters of Medicine Series, all the volumes of which have been biographies of great interest. In addition to being a story of a life, this volume is a page in the history of physiology, a page, too, which is concerned in the very birth of physiology as we know it at the present day. In a quiet out-of-the-way spot in the old province of Beaujolais, Claude Bernard was born on July 12, 1813, and here he passed his early years as a member of a family whose aspirations scarcely went beyond securing their daily bread. Then we find him in his teens engaged as assistant to a pharmaceutical chemist at Lyons, dispensing medicines and carrying them to the clients. Soon, however, his ambition outgrew the shop; he was drawn to the theatre, he wrote a play, and then he determined to seek his fortune in Paris. When just twenty-one years of age he arrived in Paris with the manuscript of his play. But on showing this to Girardin, then Professor at the Sorbonne, instead of being encouraged to devote himself to literature, medicine was suggested as a career, and taking this advice Bernard at once entered upon his medical studies. Hard, however, was the struggle described in the chapter devoted to these early days, a chapter which ends with his appointment as Magendie's *preparateur* at the College de France. At this point the author turns aside from biography to give a capital account of the position of physiological science at the time when Claude Bernard appeared on the scene, and in this and the succeeding chapters we find a masterly outline of the growth of physiological knowledge during the period dealt with, more especially, of course, in regard to those matters by the investigation of which Bernard gained undying fame. The headings of the chapters well describe the character of the book. Thus we have "Early Labours," "Physiological Science," "Glycogen," "Vaso-motor Nerves," "Other Discoveries," "His Later Writings," and "His Latter Years." Most of it is physiological. Still, it is a very readable kind of physiology, and is by no means written exclusively for physicians or even for scientific readers. The author draws attention to the brilliancy of the discoveries made by Bernard in the earlier part of his career, and contrasts with them the meagreness of the appliances which were then at his disposal. "The great truths which it was given him to lay bare were not reached by the help of easy circumstances and ample opportunities for inquiry such as fall to the lot of at least most of the young men of science of to-day." Comparing Bernard's surroundings at the commencement of his career with those of the young man who at the present time desires to devote himself to scientific work, "the latter, at least in most cases, finds in some way or other access to a well-furnished laboratory, in which the path of investigation is made smooth—perhaps, in some cases, too smooth—for him, in which adequate apparatus is placed at his disposal, and in which he is at once trained in the technique of inquiry and guided in his thoughts" by a wise and experienced master. "None of these things came to Bernard." Yet how he succeeded! Perhaps, after all, by making the path of the investigator easy we do but embarrass the true inquirer by surrounding him with too great a crowd of kid-gloved competitors for fame.

Although Professor Foster deals chiefly with the physiological investigations undertaken by the subject of his memoir, he does not neglect the more domestic side of his life, in which he does not seem to have achieved the same success as attended his efforts in the laboratory. He had unhappily married a wife who was in no way a helpmeet to him. "Her's was a nature too commonplace to rise to any sympathy with his intellectual aspirations; she was not prepared, as he was, to live laborious days on narrow means in order that the world might be the richer for the truths which he by patient toil might reap. . . . She thought that the life of a successful practitioner, riding to-day in his carriage and to-morrow forgotten, was far more to be desired than the life of a student whose present lay in obscurity and whose future could not be foretold." Who knows how far the life-long devotion to science which characterised Bernard's career may not have been due in his case, as it has been in others, to being denied the pleasing distractions of a happy domestic life. At any rate in his latter years he lived alone, for dissensions with his wife had led to an early separation, and his two daughters, his only children, were also estranged from him. "One of them indeed was so far removed from sympathy with her father's labours that she spent much of the means which fell to her in founding hospitals for dogs and cats, with a view to atoning for what she considered the crimes of vivisection which her parent had committed!" Thus to the very end he kept at work, and in the midst of unfinished work he died. It is pathetic to think of the great investigator struggling against advancing illness, bringing all his well-trained powers to bear on an inquiry of the highest importance, having the idea and the clue ready in his mind and almost mature for publication, and dying with the story still untold. When prostrated on his death-bed he said to his pupil d'Arsonval, "I am too tired and weak at this moment to explain them to you." Unfortunately, the strength and clearness of mind needed for the explanation never came back to him, and whatever his views may have been they went down with him into the grave.

